# Computational Studies of Cardiac and Skeletal Troponin

**DOI:** 10.3389/fmolb.2019.00068

**Published:** 2019-08-09

**Authors:** Jacob D. Bowman, Steffen Lindert

**Affiliations:** Department of Chemistry and Biochemistry, Ohio State University, Columbus, OH, United States

**Keywords:** troponin, molecular dynamics simulation, free energy methods, brownian dynamics, cardiac thin filament modeling

## Abstract

Troponin is a key regulatory protein in muscle contraction, consisting of three subunits troponin C (TnC), troponin I (TnI), and troponin T (TnT). Calcium association to TnC initiates contraction by causing a series of dynamic and conformational changes that allow the switch peptide of TnI to bind and subsequently cross bridges to form between the thin and thick filament of the sarcomere. Owing to its pivotal role in contraction regulation, troponin has been the focus of numerous computational studies over the last decade. These studies elegantly supplemented a large volume of experimental work and focused on the structure, dynamics and function of the whole troponin complex, individual subunits, and even on segments of the thin filament. Molecular dynamics, Brownian dynamics, and free energy simulations have been used to elucidate the conformational dynamics and underlying free energy landscape of troponin, calcium, and switch peptide binding, as well as the effect of disease mutations, small molecules and post-translational modifications such as phosphorylation. Frequently, simulations have been used to confirm or explain experimental observations. Computer-aided drug discovery tools have been employed to identify novel potential calcium sensitizing agents binding to the TnC-TnI interface. Finally, Markov modeling has contributed to simulating contraction within the sarcomere on the mesoscale. Here we are reviewing and classifying the existing computational work on troponin and its subunits, outline current gaps in simulations elucidating troponin's role in contraction and suggest potential future developments in the field.

## Introduction

**Figure 1:**
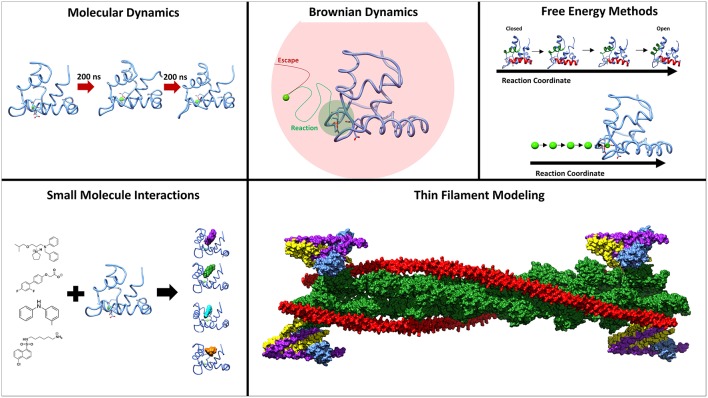
Methods employed to study the underlying molecular mechanisms of muscle contraction. Molecular dynamics and umbrella sampling have been used to study the dynamics of the hydrophobic patch region. Techniques such as BrownDye and umbrella sampling have been used to study calcium binding. Small molecules have been studied interacting with troponin C through docking and molecular dynamics. Thin filament modeling has been key to understand the larger context of muscle contraction.

Troponin (Tn) is a three-subunit protein complex that resides on the thin actin filament in muscle cells. Its three subunits, troponin C (TnC), troponin I (TnI), and troponin T (TnT) have separate roles in facilitating muscle contraction (Greaser and Gergely, 1973). TnT is anchoring the complex to the actin filament and also interacting with the protein tropomyosin. TnI has an inhibitory region that will interact with actin and inhibit the movement of tropomyosin from the myosin-binding sites on the actin filament. TnC is the calcium-binding subunit, that binds calcium in its regulatory domain which allows TnC to bind to a region of TnI known as the switch-peptide (Parmacek and Solaro, 2004). This interaction then leads to a sliding of the tropomyosin on the actin filament and exposes the myosin-binding sites for contraction to occur (Gordon et al., 2001). Understanding the interactivity between subunits within the complex is critical to understanding muscle contraction at a molecular level. An important area of study are the intrinsically disordered regions within the troponin complex that play critical roles in functional regulation (Na et al., 2016; Papadaki and Marston, 2016; Marston and Zamora, 2019). Serious health conditions, such as cardiomyopathies, have been linked to proteins within the sarcomere, and especially troponin (Hershberger et al., 2010; Seidman and Seidman, 2011). In addition to the significant experimental contributions to the study of troponin, a plethora of computational methods have been developed and utilized to study structure, dynamics and function of troponin. Here we will review computational studies of cardiac and skeletal troponin, seen in Figure 1, including molecular dynamics simulations sampling the conformational dynamics of troponin, free energy simulations used to elucidate the underlying free energy landscape of troponin, modeling of small molecule interactions with TnC, as well as troponin's role in Markov state models of sarcomere contractility.

## Molecular Dynamics Simulate the Conformational Dynamics of the Troponin Complex and Its Subunits

### Cardiac Troponin Simulations

Dynamic motions of the cardiac troponin complex and its individual subunits have been extensively studied with molecular dynamics (MD) simulations and helped elucidate the functional importance of these motions. Molecular dynamics numerically integrates Newton's equations of motion and allows for the simulation of trajectories of biomolecular atoms and molecules (Karplus and McCammon, 2002). This technique can simulate dynamics on the order of ns-ms and systems up to millions of atoms, only limited by available computational resources. A wild-type cTnT subunit was simulated to investigate the hinge dynamics and develop a model for subunit interactions (Manning et al., 2012a). Conventional MD of the N-terminal regulatory domain of cardiac TnC (cNTnC) and the cTnI-switch peptide has been used to measure the distance between key interacting residues over the course of 40 ns simulations which revealed isoform-specific interactions (Thompson et al., 2014). Continuing their investigation into the role of isoform-specific interactions, the Metzger group subsequently simulated cTnC-cTnI switch-peptide systems at various protonation states (Palpant et al., 2012). Further study of the cTnC-cTnI in complex showed that there are key structural differences between the helix 4 of TnI and the switch-peptide region (Vetter et al., 2018). The intrinsically disordered region of TnI (C-terminal domain) was simulated with cNTnC which provided evidence that the region is flexible and has structural preferences (Metskas and Rhoades, 2015). Simulation insights into the I and T subunits depend largely on their relation to the effect they have on cTnC, therefore simulations of cNTnC are critical to understanding muscle contraction at a molecular level. Simulations of cNTnC showed calcium-binding is driven, entropically, by desolvation of the calcium ion rather than structural entropy change in cNTnC (Skowronsky et al., 2013). Long timescale simulations of wild-type, calcium-bound cNTnC, on the order of 10 μs, revealed sampling of a semi-open configuration of cNTnC that is not seen in the experimental structure (Lindert et al., 2012a). As a method to enhance sampling, accelerated MD simulations were performed on the cNTnC systems which, when projected onto a PCA space, sampled the open configuration exclusively in the calcium-bound state (Lindert et al., 2012b).

Developing a model of muscle contraction through computational methods requires going from the single subunits to a complete troponin complex and even beyond. The Li lab conducted 12 ns simulations on a full cTn complex (Varughese et al., 2010). This work was able to show that calcium coordination is altered between isolated cTnC and cTnC in complex. Longer timescale simulations of the core troponin complex were subsequently performed by the Gould lab who were able to simulation for hundreds of nanoseconds (Zamora et al., 2016). This model provided insight into interactions between the subunits and can be used for further mutational studies in the Tn complex. Experimental FRET has been used by the Dong lab to restrain molecular dynamics simulations of the core of the cardiac troponin complex (Jayasundar et al., 2014). These experimental restraints provided a more direct method to relax the model of the troponin complex to a native minimum. In order to study the troponin complex in its natural environment on the thin filament, a full thin filament model was developed by the Schwartz group (Manning et al., 2011). This model was then simulated using unrestrained MD (Williams et al., 2016). These seminal simulations were able to show the influence of cTnT mutations on cTnC and provide insight on the mechanism of disease pathology.

### Fast Skeletal Troponin Simulations

To further understand the molecular basis of skeletal contraction, the fast-skeletal troponin complex (sTn) and fast-skeletal troponin C (sTnC) have been simulated using molecular dynamics. The Li group simulated a full troponin complex and demonstrated that the inter-linker region of sTnC was flexible in simulations, in contrast to what the static model suggested (Varughese et al., 2010). This work also highlighted correlated motions within the complex between the C-terminal domain of sTnC and helices of the sTnT subunit. The Lu group simulated both the core domain of sTn and an isolated sTnC subunit (Genchev et al., 2013), showing that the calcium-bound N-terminal region transitioned from the open state (which is observed in the experimentally-derived structures), to a stable semi-open configuration. Closing of calcium-bound sTnC from the open state has been detected in other MD simulations as well. The isolated N-terminal domain of sTnC was used in conventional MD simulations for 1 μs in which semi-open and open configurations were sampled, but not exclusively (Bowman and Lindert, 2018). This work further supported temporary closing of the sTnC N-terminal region, even in the calcium-bound state. The Ghosh lab modeled the missing residues of known sTnC crystal structures guided by thermodynamics (Sikdar et al., 2016). This work showed destabilization of key residues resulting from calcium binding and allowed the binding of sTnI to both domains of sTnC.

### Simulations of Disease State and Calcium Sensitivity Modulation Mutations

Mutations within the troponin complex and other sarcomeric proteins within cardiac muscle can lead to life-threatening cardiomyopathies, such as hypertrophic (HCM) and dilated (DCM) cardiomyopathy. A key use of MD is to study the dynamics of the cTn complex and its subunits in the presence of these mutations. HCM- and DCM-associated mutations that exist on the regulatory domain of cTnC have been in the focus of various conventional MD simulations. Early short simulations were run for 5 ns on the DCM-associated mutation D75Y in calcium-free cNTnC and showed that the D75Y mutation would lead to a reduction in contraction through stabilization of the closed state (Lim et al., 2008). The designed calcium sensitizing cNTnC mutation L48Q was simulated for up to 70 ns by the Regnier group (Wang et al., 2012), revealing an increase in the stability of the calcium binding site coordination and a disruption of the closed state. Additionally, the calcium desensitizing mutations L57Q and I61Q were simulated using a similar protocol as for L48Q (Wang et al., 2013). This study, also by the Regnier group, showed the destabilization of the cNTnC site II calcium-binding site caused by these mutations. Long timescale simulations of several microseconds of gain-of-function mutation V44Q and loss-of-function mutation E40A, were able to show distinct differences in the opening frequency imparted by these mutations (Lindert et al., 2012a). This modulation of opening frequency was suggested as a mechanism for calcium sensitization. An extension of this work showed that other gain-of-function and loss-of-function mutations altered the dynamic landscape of cNTnC (Kekenes-Huskey et al., 2012). This suggested that tuning the cNTnC dynamics would lead to tuning of the myofilament. Microsecond simulations of DCM-associated mutations revealed that the C-terminal cTnC mutation G159D and the N-terminal mutation D75Y both greatly reduced time spent in the open configuration of cNTnC (Dewan et al., 2016). The Tibbits group performed simulations of four HCM-associated mutations, in addition to the designed calcium-sensitizing L48Q mutation and the DCM-associated mutation Q50R (Stevens et al., 2017). These simulations showed that HCM-associated mutations destabilized the closed state of cNTnC. This result was in agreement with our simulations, showing an overall lower free energy of opening for HCM mutations and especially for the designed calcium-sensitizing mutation L48Q, and a slightly larger free energy of opening for the DCM-associated mutations (Bowman and Lindert, 2018).

Mutations that impact calcium sensitivity and lead to cardiomyopathies are also found in cTnI and cTnT and have been studied computationally. The HCM-associated cTnI mutation R145G showed little change in the overall dynamic behavior of the cTn complex compared to wild-type (Lindert et al., 2015a). This study suggested that the mutation exclusively disrupted residue-residue contacts created by phosphorylation as a mechanism for the HCM-associated mutation. This study also created a model that was ideal for studying disease-associated cTnI mutations. In addition to R145G, the cTnI mutation R21C was simulated (Cheng et al., 2015). Similarly to the R145G mutation, R21C disrupted the contacts generated by phosphorylation. The putative HCM-associated mutation P83S, studied with the same cTn model, exhibited dynamics similar to wild-type (Cheng et al., 2016). This finding agreed with the studies of R145G and R21C, in that the contacts imparted by phosphorylation were only blunted rather than completely disrupted. The Regnier lab investigated the restrictive cardiomyopathy (RCM) cTnI mutation R145W with a full cTn complex as well (Dvornikov et al., 2016). This cTnI mutant, by itself, did not alter the interactions between cTnC and cTnI. But upon addition of the phosphomimic mutations S23D/S24D, the R145W mutant disrupted the phosphorylation-mediated decoupling of cTnI and cTnC, leading to conclusion that the combination of phosphorylation and mutation lead to increased contractility. The Schwartz group has spearheaded investigations of the influence of cardiomyopathy-associated mutations on cTnT through MD. In an early iteration, residues 70–170 of murine cTnT with HCM mutations R92L and R92W were investigated in short simulations (Ertz-Berger et al., 2005). Both these mutations decreased helical stability of cTnT, as seen in disruption of hydrogen bonds, suggesting a mechanism for Tn destabilization. This work was subsequently extended employing longer simulations, on the order of 300 ps, on the same mutations (Manning et al., 2012a). Much like the previous work, this study showed decreased helical stability, and additionally suggested a mechanism of disease pathology by disrupting troponin tail and tropomyosin binding necessary for typical contraction. A full atomistic model of the troponin complex was developed for studying these HCM-associated mutations (Manning et al., 2012b). The cTnT mutations R92L, R92W, ΔE160, E163K, and E163R were found to either induce changes in the flexibility of cTnT or change the calcium affinity for the cNTnC calcium-binding site. A full cardiac thin filament model was generated for further investigation of changes in dynamics and contacts induced by these HCM-mutations (Williams et al., 2016). This work was critical in linking the cTnT mutations to allosteric effects on calcium binding within cTnC. Understanding this link has potential to target cardiomyopathies through means other than calcium-sensitivity modulating small molecules.

### Simulations of Post-translational Modifications in Troponin

Post-translational modifications, specifically PKA phosphorylation of cTnI, are crucial to function within the troponin complex which ultimately reduces calcium sensitivity and promotes muscle relaxation. Phosphomimic mutations of cTnI residues S23 and S24 to aspartic acid were found to increase the movement of the entire Tn complex while not altering the site II calcium-binding of cNTnC. These phosphomimics also led to intrasubunit interactions between the cNTnC and the inhibitory region of cTnI, a region before the switch peptide (Cheng et al., 2014). These phosphomimic mutations were then assessed in the presence of a known disease-associated cTnI mutation, R145G of cTnI, to explore its impact on a phosphorylated system. This mutation interrupted the intrasubunit interaction observed in the wild-type phophomimic simulations which suggested a mechanism for the Tn modulation (Lindert et al., 2015a). In support of the validity of the phosphomimic model, these systems were also simulated with actual phosphoserine side chains at cTnI residues 23 and 24 and no distinguishable differences between the simulations were observed. An addition of the HCM-associated cTnI mutation R21C to the complex also lowered the intrasubunit contacts observed in the wild-type system with phophomimics added (Cheng et al., 2015). In contrast to the previously described mutations, the HCM-associated cTnI mutation P83S only moderately disrupted the phosphorylation-mediated interaction between cNTnC and cTnI. This study showed that there are other possible mechanisms which are additive to the P83S mutation that led to hypertrophic cardiomyopathy (Cheng et al., 2016). These studies were further extended by the Gould group that created a full troponin complex model and simulated on the order of 750 ns to investigate phosphorylation regulation of calcium-binding (Zamora et al., 2016). Utilizing phosphoserine, instead of a phosphomimic, this work showed that the phosphorylation moved the S69 in cTnC to an out of coordination position in site II for calcium.

## Computational Studies of Calcium and TnI Binding to TnC

Techniques such as Brownian dynamics and umbrella sampling have been used to investigate the binding of calcium and TnI to TnC. Brownian dynamics is a technique that simulates a system based on an overdamped Langevin equation of motion, as opposed to Newtonian motion in MD, to study diffusion dynamics and obtain association rates for a given process (Ermak and McCammon, 1978). Browndye was utilized to estimate an on-rate for calcium for wild-type cNTnC comparable to experimentally determined values (Lindert et al., 2012b). Because this technique was able to recapitulate experimental values for wild-type, it was subsequently extended to use with disease-associated mutations of cTnC (Dewan et al., 2016). This work demonstrated that the calcium on-rate was indeed impacted by these mutations, in agreement with experimental data. Milestoning, applied to cTnC calcium binding by the Amaro group, also generated k_on_ rates in agreement with experiment (Votapka and Amaro, 2015). The Tibbits group further developed an umbrella sampling scheme to investigate calcium binding free energies in zebrafish cTnC and ssTnC at two temperatures (Stevens et al., 2016). This method has also been extended to use on cardiomyopathy-associated mutations, which was able to ascribe differences to binding energies to these mutations (Stevens et al., 2017). Steered molecular dynamics techniques used by the Schwartz group have been used to assess calcium binding to the cTn complex with cTnT mutations (Williams et al., 2016). These simulations were able to calculate the work required to pull calcium ions from cNTnC within the context of the core of cTn. Free energy perturbations from the Metzger group were able to show an increase in calcium binding free energy for acidosis states of myocytes that agreed with experimental data (Thompson et al., 2014; Vetter et al., 2018). Finally, a four state model was developed to explain, through investigation of the cTnI effective concentration, why calcium sensitivity varies from isolated TnC to the Tn complex to a full thin filament model (Siddiqui et al., 2016). In addition to the application of free energy methods to assess calcium binding, TnI binding has been explored. Both MM/PBSA (Stevens et al., 2017). and MM/GBSA (Lindert et al., 2015a). have been used to estimate the energy of binding of the cTnI-switch peptide to cNTnC. While these values did not exhibit close agreement with experimental measurements, they were still instructive in ranking scores of the approximate energy of binding for mutations of cTnI. Additionally, steered molecular dynamics and umbrella sampling methodologies have been developed to sample the free energy landscape of the troponin complex. We developed an umbrella sampling scheme for assessing the free energy of opening of the hydrophobic patch of the regulatory domain of sTnC, cTnC, and cardiomyopathy-associated mutations of cNTnC and provided insight into a potential mechanism of contraction modulation (Bowman and Lindert, 2018).

## Small Molecule Interactions With cTnC

Small molecules developed for the treatment of cardiomyopathies have been simulated bound to cNTnC to probe binding energies for these molecules through an MM/PBSA method. The Li group applied this approach to studying the well-known TnC binding molecule bepridil (Varughese et al., 2011). This work was able to show that bepridil enhanced calcium sensitivity by altering the calcium coordination residues in the isolated cNTnC, but decreased cTnC and cTnI interactions in the complex. As a result of the success of this method, it was further used in combination with drug discovery to validate calcium-sensitization of new compounds (Varughese and Li, 2011).

Treatment of the pathologies associated with diseased cardiac muscle has been of great interest. To this end, cNTnC has been a target for small molecule drug screens and drug development. High through-put virtual screens (HTVS) on clusters derived from MD simulations were performed on cNTnC. This technique was able to identify a calcium-sensitizing compound, NSC147866, from the NCI II diversity set (Lindert et al., 2015b). A significantly improved version of this screening protocol, applied to structures of cNTnC from 100 ns simulations, found two additional calcium sensitizers, NSC600285 and NSC611817, from the entire NCI database (Aprahamian et al., 2017). Employing experimental intuition, instead of blind screens, compounds that were similar in structure to diphenylamine were docked into cNTnC (Cai et al., 2016). This allowed for the identification of the calcium sensitizer, 3-methyldiphenylamine. Small molecules bound to cNTnC have also been studied with an umbrella sampling scheme to show their influence on the free energy landscape (Bowman et al., 2019). In contrast to studying the cNTnC hydrophobic patch as a target for drugs, recent work has targeted the interdomain linker between N-domain and C-domain of cTnC (Szatkowski et al., 2019). Efficacy of these drugs was measured by changing of interaction between the tropomyosin and cTnT.

## Markov Modeling Has Contributed to Simulating Contraction Within the Sarcomere on the Mesoscale

Isolated models and simulations of Tn and its subunits provide a valuable, yet small window into muscle contraction. There is, however, a need to correlate these energetics and kinetics found at the protein level to the sarcomere level. Through Markov state modeling, in which the next state depends only on the current state of the system, these individual studies can be linked together to create picture of muscle contraction. A model of these processes was created based on the calcium binding, tropomyosin movement, and then myosin binding (Campbell et al., 2010). This model accurately predicted steady-state force change. The Markov model from the Campbell group was subsequently updated to include the azimuthal angle between tropomyosin between adjacent tropomyosin chains (Sewanan et al., 2016). Addition of this angle was unaccounted for in previous models and allowed for incorporation of tropomyosin mutations into the model. An updated model was proposed that included the opening of the hydrophobic patch and binding of the cTnI switch peptide (Dewan et al., 2016). This model used data from Brownian dynamics and molecular dynamics simulations. While unable to accurately predict the impact of cardiomyopathy-associated mutations on contraction, this model was able to show that small changes in these states can ultimately alter the pCa curves at the larger scale.

## Current Gaps and Potential Future Developments in the Field

Computational methods have already made a significant contribution to our understanding of the dynamics and function of troponin. However, several gaps in simulations elucidating troponin's role in contraction remain. The accuracy of free energy calculations, particularly with respect to calcium binding affinities, is currently insufficient, as a result of inaccuracies in forcefield descriptions of calcium, non-classical electronic effects, and a lack of robust sampling of the thermodynamic ensemble. Future efforts will have to focus on more accurately predicting calcium binding affinities, probably employing longer simulations, force field optimization, polarizable force fields or even QM/MM calculations. In the context of calcium binding, but not limited to it, the behavior of the troponin complex is distinctive from that of its substituents (e.g., isolated cNTnC, isolated cTnC), challenging simulations to correctly account for those differences. In general, design of additional computational experiments that are verifiable *in vitro/vivo* will lead to more cohesion between models and experiments. Another current limitation is the disparity between physiologically-relevant millisecond-scale conformational dynamics of the contractile system and the restriction of conventional simulations to tens of microseconds, often accompanied by simulation of very small sections of the contractile machinery. The Schwartz group has paved the way for extending the size of simulations to the thin filament and it is our prediction that the field will follow in the years to come. An alternative route to obtaining contractile information on the mesoscale are the Markov models developed by Campbell and coworkers. Future work will likely focus on obtaining additional model input from computational simulations, such as the accurate predictions of calcium-binding affinities discussed above, as opposed to experimental measurements.

## Author Contributions

All authors listed have made a substantial, direct and intellectual contribution to the work, and approved it for publication.

### Conflict of Interest Statement

The authors declare that the research was conducted in the absence of any commercial or financial relationships that could be construed as a potential conflict of interest.
